# Studies Based on Preparation, Physical Characteristics, and Cellular Pharmacological Activities of Thin PLGA Film Loaded with Geniposide

**DOI:** 10.1155/2014/352423

**Published:** 2014-02-12

**Authors:** Haiyan Zhang, Hao Liu, Nan Huang, Ya He, Tingting Lei, Xin Wang, Ming Yang, Guangming Luo

**Affiliations:** ^1^Key Lab of Advanced Materials Technology of Ministry of Education, Southwest Jiaotong University, Chengdu 610003, China; ^2^Key Laboratory of Modern Preparation of TCM, Ministry of Education, Jiangxi University of Traditional Chinese Medicine, Nanchang 330004, China

## Abstract

In this primary study, thin polylactic-co-glycolic acid (PLGA) film loaded with geniposide was first prepared and demonstrated on both physical and pharmacological aspects for its potential application on drug-eluting vascular stents. Physical parameters of geniposide-loaded thin film, such as crystal structure, molecular spectral characteristics, and release behavior in the whole process were detected. From X-Ray diffraction, the characteristic peak of crystal geniposide disappeared on geniposide-loaded PLGA film (GLPF) after it formed, which meant there was no agglomeration phenomenon, as geniposide was distributed in the form of single molecule. According to scanning electron microscopy (SEM) figure, the GLPF was more flat and uniform with better compactness. It inferred that release behavior of geniposide at the early stage (0~15 d) was in the form of free diffusion. Carrier PLGA began to degrade 15 days later, so the residual geniposide was also dissolved. Cellular pharmacological effects of geniposide on endothelial cells (ECs) and smooth muscle cells (SMCs) were also demonstrated on GLPF. 5% and 10% (w/w) geniposide-loaded PLGA (60 : 40) membrane indicated its significant effect on ECs promotion and SMCs inhibition. All provided feasible evidences for the development of new geniposide-coating vascular stent using PLGA as carrier.

## 1. Introduction

In recent years, since vascular stent is successfully applied in clinical surgery, practitioners have to consider more complication problems caused by it such as endothelial damage, thrombosis, and vascular restenosis. As the vascular endothelial injury may happen during the inserting, moving, or even supporting processes of stent, injury would promote vascular inflammation and inspire platelet aggregation. Both smooth muscle cell proliferation (VSMC) induced by series of inflammation response and thrombosis induced by platelet aggregation development would result into intimal hyperplasia finally. Both of intimal hyperplasia and thrombosis would lead to restenosis. To reduce the incidence of vascular restenosis, scientist attempted to spray some biodegradable drug-loaded films on the stents [1–3]. The loaded drug is often controlled to release by its carrier, so it would only be able to affect the partial blood vessel where it is inserted but not cause severe systemic toxicities or adverse events. Therefore, drug-coating vascular stent (DES) gradually became a very ideal design for efficient prevention for neointimal proliferation and vascular restenosis [4–6].

Nowadays, the mainstream prevention strategies for vascular restenosis depend on the prohibition of VSMC proliferation and the realization of early reendothelialization and antithrombosis. Geniposide, the main component of traditional Chinese herbal medicine *Gardenia jasminoides* Ellis, is just fit for all of those requirements, as it plays an important role in the activities of antiinflammation [7, 8], antioxidation [9, 10], anticoagulation [11], antithrombosis [12], cardiovascular protection and cerebral nerve repair [13, 14]. Various studies also inferred that *Gardeniae Fructus* extract exhibited positive effect on promoting endothelial cells (ECs) proliferation, protecting endothelial function injured by variety of factors, and meanwhile, showed no obvious effect on smooth muscle cells (SMCs) [15–17]. Its worth noting that those physiological and pathological disorders are closely associated with each other, as endothelial injury can cause thrombosis, which also can make endothelial injury more severe conversely. Therefore, it is believed that geniposide may play important role on the prevention of thrombosis and vascular restenosis for its multiple pharmacological activities and it can be fit for loading on DES theoretically.

The coating of DES commonly requires not only excellent biological compatibility, but also its own physical characteristics, such as good smoothness, compactness, and thermal stability. Polylactic-co-glycolic acid (PLGA), a kind of biodegradable polymer carrier material, was utilized as drug carrier in nanomaterials preparation or drug-loaded vascular scaffolds for a long time [18–20]. In this study, the related preparation process of film loaded with geniposide was studied. The relative physical characteristics of film were detected and estimated as well, including thickness, surface topography analysis of scanning electron microscopy (SEM), structure analysis of X-ray diffraction (XRD), surface structure analysis of Fourier transform infrared spectroscopy (FTIR), thermodynamic behavior analysis (TG), and so forth. After that, its pharmacological effects on ECs and SMCs were also evaluated. All of them try to exam the primarily practical feasibility of geniposide-loaded PLGA film (GLPF) for DES.

## 2. Methods and Steps

### 2.1. Materials and Equipment

PLGA60000 (LA : GA = 50 : 50), PLGA60000 (LA : GA = 60 : 40), and PLGA60000 (LGA : GA = 75 : 25) were purchased from Shenzhen Eco Biomaterial Co., Ltd; geniposide (purity degree of 98.1%, Shanghai Jingsen Biology Science & Technology Co., Ltd); silicon chips (Beijing Xinxing Braim Technology Co., Ltd); both chloroform and ethanol were AR grade; methanol and phosphate were HPLC grade; and ddH_2_O and PBS were prepared by ourselves.

Analytical balance (Sartorius Scientific Instruments (Beijing) Co., Ltd); thermostatic magnetic stirrer (78HW-1, Yitong Electronics Co., Ltd.); vacuum drying oven (DZF-6050, Shanghai Cimo Medical Devices Manufacturing Co., Ltd); ultrasonic cleaner (KQ-250VDB, Kunshan Ultrasonic Instruments Co., Ltd); constant temperature shaker (QYC-200, Shanghai Fuma Laboratory Instrument Co., Ltd); high performance liquid chromatography (LC-2010AHT, Shimadzu Corporation); octadecylsilyl column (5 *μ*m, 4.6 mm × 150 mm, Dalian Elite Analytical Instruments Co., Ltd); step profiler (XP-2, Ambios Technology Inc.); multifunctional X-ray diffractometer (BeDe D1 System, Britain Bede Scientific Instruments Ltd); scanning electron microscope (JSM-6701F, JEOL Ltd.); diamond DSC (PerkinElmer Inc.); electronic balance (FA1104, Shanghai Minqiao Medical Appliances Co., Ltd); and Fourier transform infrared spectroscopy (VERTEX 70, Bruker Corporation), UV-Vis spectrophotometer (U-3000, Shimadzu Corporation).

### 2.2. Preparation of PLGA Membrane Loaded with Geniposide

#### 2.2.1. Screening of Geniposide Loading Dosage

The different proportions of geniposide and PLGA greatly impact the formability of membrane. According to the different loading ratios (10%, 15%, and 20% w/w) of geniposide (10 mg, 15 mg, and 20 mg) in membrane, different amounts of PLGA (90 mg, 85 mg, and 80 mg) were weighed and dissolved in 5% (w/v) chloroform solution by the magnetic stirrer stirring to be transparent. Based on the optimal ratio of chloroform and ethanol, geniposide was dissolved in certain volume of ethanol and then ethanol and chloroform were mixed together.

#### 2.2.2. Membrane Preparation

270 mg of PLGA (PLGA (50 : 50), PLGA (60 : 40), and PLGA (75 : 25)) in a molecular weight of 60,000 Da was weighted to prepare 5% (w/v) concentration of PLGA in chloroform, respectively. They were stirred by magnetic stirrer slowly after swelling overnight. 90 mg geniposide was weighed and dissolved in ethanol to be 50 mg/mL. Total solution of 50 mg/mL geniposide was added separately into PLGA (50 : 50), PLGA (60 : 40) and PLGA (75 : 25) with continuously stirring. The blank controls of PLGA (50 : 50), PLGA (60 : 40), and PLGA (75 : 25) polymers were also prepared. Then cosolvent was stirred and degassed by ultrasonic, and finally the same volume of solution was poured into the precleaned glass dishes, in which there were prewashed dry silicon chips. After solvent was naturally evaporated, silicon chips with drug coating were dried under vacuum for 48 h.

### 2.3. Physical Characterization of Geniposide-Loaded Membrane

#### 2.3.1. Determination of Membrane Thickness

After selecting several local areas of blank or drug-loaded PLGA membrane, step profiler was utilized to test the average membrane thickness.

#### 2.3.2. SEM Analysis of Surface Morphology

The micromorphology and composition structure of polymer membrane is very important to its surface properties. To study the morphology and structural characterization of polymer membrane, scanning electron microscope (SEM) is an effective mean. The magnification of scanning electron microscope is about 5~40000. Before measurement, the membrane must be dried and sprayed with gold.

#### 2.3.3. Structure Analysis by XRD

The multifunctional X-ray diffractometer (Model: BeDe D1 System) was used to record X-ray diffraction pattern of blank/drug-loaded PLGA membrane in different proportions. CuK*α* rays were utilized with the scan angle (2*θ*) of 10°~80° and scanning speed of 10°/min.

#### 2.3.4. FTIR Analysis of Molecular Groups

To detect the molecular groups of drug-loaded membrane and complex situation between drug and carrier, FTIR was used to scan the surface of membrane within wavelength range of 4000 cm^−1^~400 cm^−1^.

#### 2.3.5. TG Behavior Analysis

The prepared drug-loaded membranes and geniposide was placed into thermal gravimetric analysis systems with heating rate of 10°C/min and the change curve of temperature from 25°C to 250°C. The weight change curve of samples along with different temperature was recorded.

### 2.4. In Vitro Release Behavior of Drug Film

#### 2.4.1. Basic Release Situation of Geniposide

The drug membranes coated on the substrate of silicon chips were put into conical flask with piston. 50 mL PBS solution was added as release medium (pH = 7.4). And then they were placed in a shaker with constant temperature of 37°C and oscillation frequency of 100 rev/min. After oscillation started, 1 mL liquid was drawn out after the intervals per day, while 1 mL of fresh PBS was added as well. The whole testing period was about one month.

#### 2.4.2. Measurement of Geniposide

The liquid of geniposide releasing was diluted into certain concentration within the linear range of geniposide; it was measured by UV at the absorbance of 238 nm. According to the standard curve, concentrations of geniposide could be calculated.

### 2.5. The Cellular Pharmacological Effects on ECs and SMCs

The GLPFs (containing 1%, 5%, and 10% geniposide, w/w) were dissolved in cosolvent firstly, respectively, and then the solvent was change into culture solution. The supernatant was separated and added into 96-well plate as drug intervention. (1) For its influence on ECs proliferation, the cell culture was stopped 4 h later after adding MTT solution (5 g/L, medium : MTT = 10 : 1). The solution in well plates was abandoned and changed into DMSO solution (MTT : DMSO = 1 : 10). After 10 min vibration in low speed, 200 *μ*L solution (*n* = 6) was removed into new 96-well plate for the detection of optical density (OD) value at 490 nm wavelength by microplate reader. (2) For its influence on SMCs proliferation, the cell culture was stopped 2 h later after adding CCK-8 reagent (CCK-8 : DMEM = 1 : 9). After 10 min vibration in low speed, 200 *μ*L solution (*n* = 6) was removed into new 96-well plate for the detection of OD value at 450 nm wavelength by microplate reader.

### 2.6. Statistical Analysis

Results were shown in the form of mean ± SEM. Data were analyzed by one-way ANOVA, followed by student's two-tailed *t*-test for comparison between two groups. *P* < 0.05 indicates statistical significance.

## 3. Results and Discussion

### 3.1. Screening of Cosolvent and Loaded Dose

The final selection of cosolvent was chloroform : ethanol = 1 : 10. The results of different geniposide-loaded doses (10%, 15%, and 20%, w/w) showed that only 10% geniposide content resulted in no precipitation during membrane formation with good compatibility.

### 3.2. Characterization Results of Membrane

#### 3.2.1. Detection of Film Thickness

Randomly selecting some points of both blank and GLPF, their thicknesses were tested by step profiler and the average thickness was calculated as well. The mean value of blank PLGA film was 40.2 *μ*m, while that of GLPF was 40.4 *μ*m.

#### 3.2.2. Surface Topography Analysis by SEM

**Figure 1:**
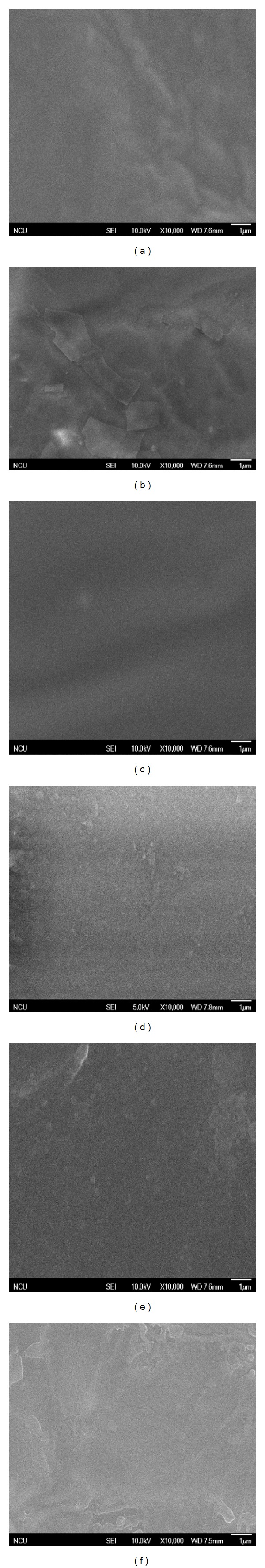
SEM images of blank membranes and geniposide-loaded membranes on PLGA (50 : 50), and PLGA (60 : 40), PLGA (75 : 25) ((a) PLGA (50 : 50) blank membrane; (b) PLGA (50 : 50) drug-loaded membrane; (c) PLGA (60 : 40) blank membrane; (d) PLGA (60 : 40) drug-loaded membrane; (e) PLGA (75 : 25) blank membrane; (f) PLGA (75 : 25) drug-loaded membrane) (10000x).

The profiles of both blank membrane and geniposide-carried membrane in the same magnification (10000×) under the SEM photograph were showed in Figure 1. As (b), (d), and (f) were for three different types of blank PLGA membrane, the smooth and flat degrees of (b) and (f) surfaces were relatively poor. There were still crystal blocks that existed on the surface of membrane (b), which was also proved by Figure 1(b). The surface of (f) was irregular. Compared with Figures (b), (f), and (d) surface was smoother with better density.

In the preexperimental results of doses screening with 15% geniposide loading on three types of PLGA membranes, the precipitation dosage of geniposide in PLGA (60 : 40) carrier membrane was minimal with better appearance than other two membranes containing PLGA, one of which was PLGA (75 : 25) carrier membrane with more bubbles and precipitation of geniposide particles, followed by PLGA (50 : 50) membrane containing a little amount of bubbles and geniposide particles. Furthermore, PLGA (60 : 40) did not contain bubbles with only minimal particles precipitation. It meant the compatibility between PLGA (60 : 40) and geniposide was better without damage to the membrane.

#### 3.2.3. XRD-Ray

**Figure 2:**
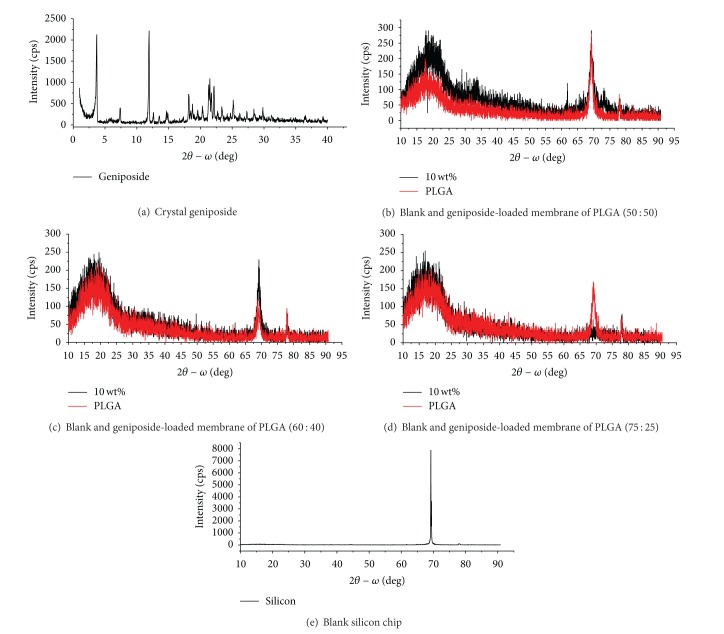
XRD patterns of (a) crystal geniposide, blank, and geniposide-loaded membrane of (b) PLGA (50 : 50), (c) PLGA (60 : 40), (d) PLGA (75 : 25), and (e) blank silicon chips.

X-Ray diffraction was shown in Figure 2, (a) was the crystalline structure of geniposide, and its main characteristic absorption peaks were at the range of 2*θ* = 2.5°~12.5°, which disappeared in all the PLGA membranes, indicating that there was no agglomeration phenomena but in the state of amorphous. The picture of (e) was XRD pattern of blank silicon chip with the characterized peaks at the position of 2*θ* = 69°. The same peak at the position of 2*θ* = 69° appearing in the (b), (c), and (d) diagram was the absorption peak of silicon substrate sheet. In the XRD pattern of (b), there were absorption peaks of crystal geniposide in films. There were no characteristic absorption peaks of geniposide in GLPFs (c) (d) compared with (a), which also meant it existed in the form of solid solution with better stability except (b).

#### 3.2.4. FTIR Analysis of Surface Structure

**Figure 3:**
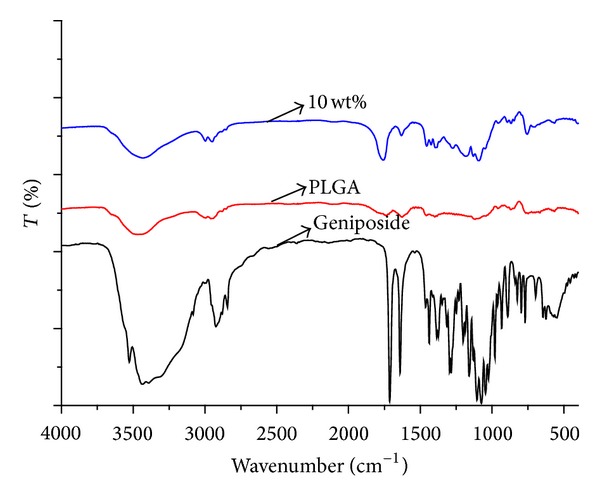
FTIR diagram of geniposide, PLGA composite and GLPF.

Figure 3 was the infrared spectrums of blank membrane and drug-loaded membrane of PLGA (60 : 40). The peak with the wave number of about 3490 cm^−1^ had a stretching vibration peak of –OH. It might indicate that there was –OH at the end of the chain during the polymerization of PLGA. The absorption intensity of geniposide-loaded membrane at this position did not increase compared with blank PLGA membrane. The bimodal around 2914 cm^−1^ was the stretching vibration peaks of C–H in –CH_2_. The peak at the position of 1770 cm^−1^ had a vibration peak of –C=O. There was no obvious move and change of corresponding peaks comparing geniposide with geniposide-loaded membrane. All these results suggested that there was no other new chemical bonding created. Maybe there was no chemical reaction between PLGA and geniposide and geniposide might exist in PLGA membrane in the state of physical dispersion maintaining the respective properties of geniposide and PLGA.

#### 3.2.5. Thermodynamic (TG) Acts of Drug-Loaded Membrane

**Figure 4:**
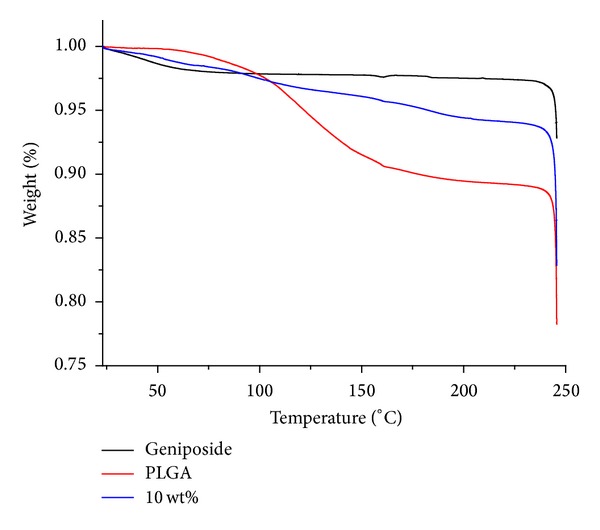
TG acts of geniposide composites, PLGA, and geniposide-loaded composite membranes.

As it shown in Figure 4, pure PLGA began to lose weight significantly at 97°C, while geniposide started to lose weight at 240°C. The weight loss rate of GLPF was in between geniposide and PLGA. The relative thermal stability order of these three substances was: PLGA < 10 wt% < geniposide. The results suggested that geniposide loading improved the thermal stability of PLGA membrane.

### 3.3. Basic Release Situation of Geniposide

#### 3.3.1. Determination of UV Absorption Wavelength of Geniposide

5.39 mg standard geniposide was weight and added into methanol preparing 106.8 *μ*g/mL solution. Standard geniposide solution was tested by UV-visible spectrophotometer in wavelength range of 200~600 nm to obtain the absorption curve. The result showed that geniposide had a maximum absorption at 238 nm; therefore, 238 nm was chosen as measurement wavelength of geniposide.

#### 3.3.2. UV Methodology Establishing

5.34 mg standard geniposide was precisely weight and put into 50 mL volumetric flask, diluted to the mark with methanol. Ultrasonic dissolution was also conducted. Standard geniposide solutions 0.1, 0.2, 0.4, 0.5, 0.6, and 0.7 mL were precisely drawn and put into 5 mL volumetric flask. Methanol was added to the mark. Volumetric flask was shook and standing for 10 min. Using methanol solution as blank reference, the absorbance of geniposide at 238 nm was measured. Concentration (*μ*g·mL^−1^) as abscissa and absorbance as ordinate, standard curve regression equation was calculated as *Y* = 0.024*X* − 0.0072, *R* = 0.9998. The result showed that the absorbance of geniposide was in a good linear relationship with concentration in the range of 9.99 *μ*g/mL~29.97 *μ*g/mL. The average recovery was 102.82% with 4.4% of RSD (*n* = 5). The precision test showed that intraday RSD was 1.29% and interday RSD was 1.83%.

#### 3.3.3. Basic Release Situation of Geniposide

**Figure 5:**
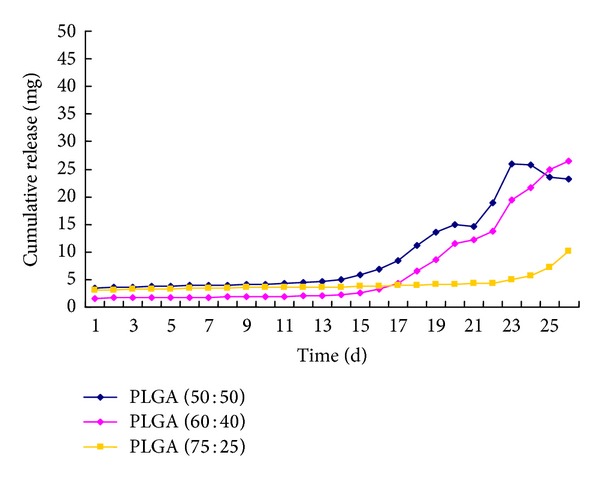
Drug release profile of GLPF.

Using sampling time as the abscissa and the calculated cumulative release of geniposide as the ordinate, release curves of geniposide in three different geniposide-coated PLGA membranes were shown in Figure 5. Release behaviors of geniposide in three carrier materials were relatively flat in the first 15 days, subsequent release was cumulatively increased.

**Figure 6:**
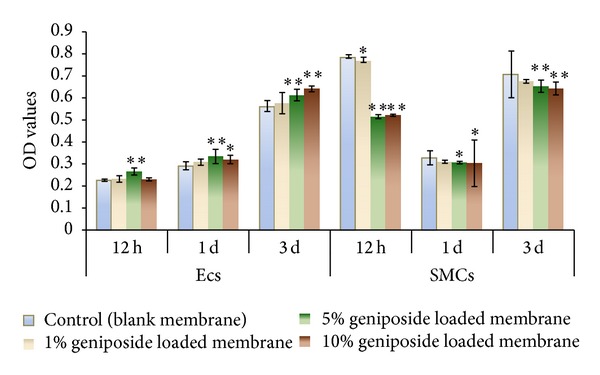
The effects of GLPF (60 : 40) on ECs and SMCs proliferation (*n* = 6).

During the first 15 days of geniposide release, the sequence of release speed was PLGA (60 : 40) < PLGA (75 : 25) < PLGA (50 : 50). For the strong hydrophilic and crystal geniposide existing of PLGA (50 : 50), its degradation rate was much higher than the others. For the rough surface morphology, release rate in PLGA (75 : 25) was also higher than PLGA (60 : 40). It demonstrated biocompatibility between geniposide and PLGA was mainly dominant in the release behavior. Although drug release in PLGA (60 : 40) in the first 15 days was slow compared with others, the release dosage still reached its effective therapeutic dosage according to the post-studies on its cellular pharmacological effects (Figure 6). Considering the dose of loaded drug in practical application is very limited, drug release mainly focuses on the first 15 days, so PLGA (60 : 40) is relatively good for slow release in the form of free diffusion—keeping low partial blood concentration for long term. Since stent-associated vascular restenosis generally occurred within 2-3 weeks after implantation into human body, so geniposide release behavior in these prepared films might basically meet the clinical needs.

15 days later, release rate in geniposide was elevated due to more micropores appeared on the surface of film after the previous geniposide release and PLGA itself starting degrading. The microporous structure of the carrier made it easier to absorb water and swell, and more micropores exposure also increased the contact area of drug and solvent, so drug was more easily released from the carrier material, accelerating membrane degradation, which might be the main mechanism of geniposide release in later period. Geniposide release in PLGA (50 : 50) was the fastest, but the release in PLGA (75 : 25) was very gentle within 20 days, which meant that PLGA (75 : 25) itself was relatively hard to degrade. In clinical, the situation of drug depleted without complete PLGA (75 : 25) degradation might occur, which might become the risk of inducing thrombosis. It indicated that release behaviors were affected by many factors, not only interrelated with the types of carrier material and diffusion coefficient of drugs, but also closely related to the natural properties of drug itself, the compatibility of drug and carrier material and the dispersed state of drug in carrier material [10].

### 3.4. The Effects of GLPF (60 : 40) on ECs and SMCs

In the cell culture studies of ECs and SMCs for 1 d and 3 d (Figure 6), the 5% and 10% (w/w) geniposide loaded membranes showed significant promotion activities on ECs proliferation and obviously inhibited SMCs proliferation (*P* < 0.05). Only 5% geniposide resulted in EC's remarkable number increase after 12 h culture (*P* < 0.01). In the other hand, 5% and 10% geniposide played similar inhibition effect on SMCs proliferation (*P* < 0.05). It meant that the cellular pharmacological effects of geniposide might not be dose-dependent. On contrast, middle dose of 5% geniposide might play stronger activity on ECs proliferation than others in the early stage.

## 4. Conclusion

For the significant solubility differences between geniposide and PLGA polymer carrier without relevant reports before, the screening of cosolvent for geniposide and PLGA was the key point for the whole formation process of membrane. In the preparation process of membrane, the evaporation rate of solvent impacted membrane thickness greatly, so strict control on the evaporation rate of solvent was very important. As the degradation of polymer carrier was influenced by the thickness and smoothness of membrane, the uniformity and smoothly flat was required for membrane. Thats why it was necessary to control the hardness, thickness, surface smoothness, and apparent shape of membrane with high requirements. Therefore, the entire deposition process of membrane formation should be strictly controlled. All these physical characterizations of polymer membrane (XRD, FTIR, TG, SEM) provided some experimental evidences for the animal or even clinical application of GLPF stents. It might offer another possibility in the form of drug-film stents for the treatment of stent-related vascular restenosis. Meanwhile, it was also a significant innovation for new types of drug-film stents.

Based on the results above, the following conclusions can be drawn: (1) by results of SEM, it could be learnt that geniposide did not destroy the shape of PLGA membrane after it formation, but kept all characteristics of vector itself such as smoothness, integrity, and compactness and so on; (2) via results of XRD, it could be learnt that there were few crystal peaks of geniposide in GLPF, suggesting that the carrier PLGA inhibited the crystallization of geniposide; (3) from results of FTIR, it showed that there was no chemical reaction between drug and carrier in drug-loaded membrane, but in the form of physical blending keeping the individual natures of drug and PLGA; (4) results of thermodynamic behavior showed geniposide-PLGA membrane system was more stable than PLGA carrier itself; (5) the overall trend of drug release behavior in prepared three GLPFs was good with no obvious burst release behavior, especially PLGA (60 : 40); (6) the carrier material PLGA had large impact on drug release, generally drug release was controlled by diffusion at the beginning, when the molecular weight of PLGA was reduced to a certain critical value, and the drug release is mainly due to degradation itself; and (7) Drug release in geniposide-loaded PLGA (60 : 40) film was associated with the development process of vascular restenosis on both its release dose and sustained release cycle; therefore, the prepared geniposide-loaded PLGA (60 : 40) film in this study might have better therapeutic effect on stent-associated vascular restenosis with good drug release behavior.
